# Network pharmacology and in vitro studies reveal the pharmacological effects and molecular mechanisms of Shenzhi Jiannao prescription against vascular dementia

**DOI:** 10.1186/s12906-021-03465-1

**Published:** 2022-02-02

**Authors:** Danfeng Tian, Qiang Gao, Ze Chang, Jingfeng Lin, Dayong Ma, Zhenyun Han

**Affiliations:** 1 Beijing University of Chinese Medicine, No.11 East road, North 3rd Ring Road, Beijing, 100029 China; 2 Neurology Department of Dongzhimen Hospital, Beijing University of Chinese Medicine, No.5 Haiyuncang, Dongcheng District, Beijing, 100700 China; 3 Shenzhen Hospital, Beijing University of Chinese Medicine (Longgang), No.1 Dayun road, Sports New City Road, Shenzhen, 518172 China

**Keywords:** Shenzhi Jiannao prescription, Network pharmacology, Vascular dementia, Pharmacological effects and molecular mechanisms, In vitro studies

## Abstract

**Background:**

Shenzhi Jiannao (SZJN) prescription is a type of herbal formula adopted in the management of cognitive impairment and related disorders. However, its effects and related regulatory mechanisms on vascular dementia (VD) are elusive. Herein, network pharmacology prediction was employed to explore the pharmacological effects and molecular mechanisms of SZJN prescription on VD using network pharmacology prediction, and validated the results through in vitro experiments.

**Methods:**

Through a search in the Traditional Chinese Medicine Systems Pharmacology Database and Analysis Platform (TCMSP) database, chemical composition and targets for SZJN prescription were retrieved. The potential targets for VD were then obtained from the GeneCards and DisGeNET databases. The network was constructed that depicted the interactions between putative SZJN prescription and known therapeutic targets for VD using Cytoscape 3.7.1. Analysis of protein-protein interaction was achieved via STRING 11.0 software, followed by Gene Ontology (GO) functional enrichment and Kyoto Gene and Genome Encyclopedia (KEGG) pathway analyses. To validate the computer-predicted results, in vitro experiments based on an excitotoxic injury model were designed using glutamate-exposed PC12 cells, and treated with varying concentrations (low, 0.05; medium, 0.1 and high, 0.2 mg/mL) of SZJN prescription. Cell viability and cell death were detected using the IncuCyte imaging system. Moreover, the expression profiles of Caspase-3 were analyzed through qRT-PCR.

**Results:**

Twenty-eight potentially active ingredients for SZJN prescription, including stigmasterol, beta-sitosterol, and kaempferol, plus 21 therapeutic targets for VD, including PTGS2, PTGS1, and PGR were revealed. The protein-protein interaction network was employed for the analysis of 20 target proteins, including CASP3, JUN, and AChE. The enrichment analysis demonstrated candidate targets of SZJN prescription were more frequently involved in neuroactive ligand-receptor interaction, calcium, apoptosis, and cholinergic synaptic signaling pathways. In vitro experiments revealed that SZJN prescription could significantly reverse glutamate-induced cell viability loss and cell death, and lower the levels of Caspase-3 mRNA in glutamate-induced PC12 cells.

**Conclusions:**

Collectively, this study demonstrated that SZJN prescription exerted the effect of treating VD by regulating multi-targets and multi-channels with multi-components through the method of network pharmacology. Furthermore, in vitro results confirmed that SZJN prescription attenuated glutamate-induced neurotoxicity.

## Background

Vascular dementia (VD), as the main type of dementia in Asia, is mainly the result of vascular risk factors such as atherosclerosis, hypertension and micro-strokes [1]. VD accounts for about 30% of dementia in Asia, and the incidence rate in China is 1.1 to 3% [2–4]. With aging, the incidence of VD is doubled every 5.3 years, which brings about a large burden on society and patients’ families [2, 5]. The currently used primary preventive medications, including cholinesterase inhibitors and excitatory amino acid receptor antagonists against vascular cognitive impairment have very limited efficacy. Thus far, there are no curative treatments available and therefore, development of therapy for VD has become an essential but unmet need. A large number of experimental studies and clinical observations have confirmed that Chinese medicine has unique advantages in treating VD. Various empirical prescriptions and active ingredient extracts have shown clear neuroprotective effects on VD [6–8].

Shenzhi Jiannao (SZJN) prescription, the national new formula in China (ZL201010249665.8), is a classical herbal formula for the treatment of VD based on the clinical practice. This prescription is mainly composed of *Panax ginseng* C.A.Mey. (Ginseng), *Anemarrhena asphodeloides* Bunge (Anemarrhenae), and *Paeonia veitchii* Lynch (Paeoniae rubra). In previous pharmacological study, it has been found that SZJN prescription improved cerebral ischemia in VD rats, reduced the levels of glutamate and γ - aminobutyric acid in the anterior cortex, and increased acetylcholine content [9]. Moreover, SZJN prescription was associated with significantly low expression of N-methyl-D-aspartate receptor 1 (NMDAR1) in the brain of rats with vascular dementia. Furthermore, administration of this prescription impeded the loss of hippocampal neurons, neuronal damage, degeneration, and necrosis; this consequently improved cognitive function [10, 11]. The effect of SZJN prescription on the central cholinergic system improves the cognitive ability of mice with acquired memory disorder through inhibition of AChE activity [12]. However, reports about the therapeutic effects and mechanisms of action of SZJN prescription on VD are scanty. The molecular mechanism of SZJN prescription has not been certainly clear.

**Fig. 1:**
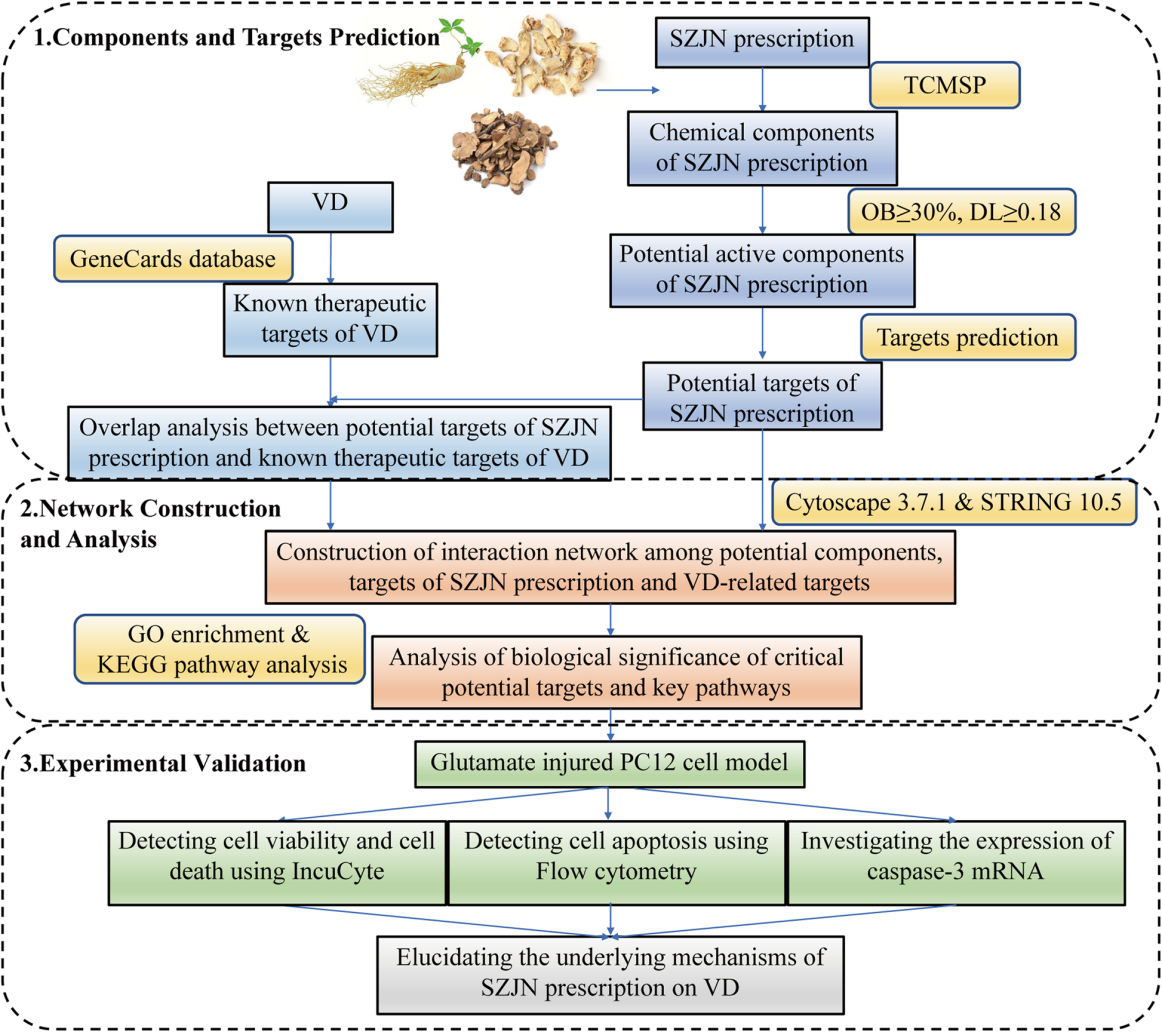
The flowchart of network pharmacology-based strategies for investigating the mechanisms of acting on VD

Network pharmacology approaches have been proven to be a powerful way for mechanistic exploration of Chinese medicine from the perspective of multicomponents, multitargets, and multichannels, which is consistent with the holistic view of Chinese medicine [13, 14]. The aim of this study was to explore the underlying molecular mechanisms of SZJN prescription in treating vascular dementia by using network pharmacology approaches and its protective effect on glutamate-induced neurotoxicity by in vitro experiment based on network pharmacology prediction. These findings provide a new scientific basis for the future development of SZJN prescription. A flowchart of the experimental procedures of this study is outlined in Fig. 1.

## Methods

### Collection, retrieval, and screening of main chemical components of SZJN prescription

To determine and collect the chemical components of the three herbs contained in SZJN prescription, a search was performed from the Traditional Chinese Medicine Systems Pharmacology Database and Analysis Platform database (TCMSP, http://ibts.hkbu.edu.hk/lsp/tcmsp.php) manually. Subsequently, compound names and their molecular structures were verified in the PubChem database (https://pubchem.ncbi.nlm.nih.gov/). Oral bioavailability (OB) ≥ 30% and drug-like (DL) ≥ 0.18 parameters were incorporated in the TCMSP database as drug screening conditions for the compounds.

### Predicting SZJN prescription chemical components targets, VD significant targets and SZJN prescription therapeutic targets

The main chemical components in SZJN prescription were determined and individually matched with potential targets according to TCMSP. First, names of the predicted target proteins were transformed into gene names via the UniProt database (http://www.uniprot.org/), then target species were selected as “humans”. Targets related to VD were collected from two databases. One was the GeneCards (https://www.genecards.org/), which is a searchable, integrative database that provides comprehensive, user-friendly information on all annotated and predicted human genes [15]. The other database was the DisGeNET (http://www.disgenet.org/), which is a comprehensive gene-disease association (GDA) database that provides the current knowledge of human genetic diseases [16]. A general search for all genes was conducted using the keywords “vascular dementia”, then retrieved from the GeneCards and DisGeNET databases. An intersection between SZJN prescription targets and VD significant targets was made to obtain the therapeutic targets for SZJN prescription for subsequent studies.

### Network construction and analysis

Using the Cytoscape 3.7.1 software, prediction results of potential targets for SZJN prescription were acquired, and a network of herbs-putative active components-putative therapeutic targets was constructed. The network topology analysis was achieved using a plugin in Cytoscape 3.7.1, after which a protein-protein interaction (PPI) network of the SZJN prescription was constructed via the STRING 10.5 (https://string-db.org) software.

### Analysis of Gene Ontology (GO) function enrichment and Kyoto Genes and Genomes Encyclopedia (KEGG) pathway enrichment

GO functional enrichment and KEGG pathway enrichment analyses were conducted using the David v 6.8 database (https://david.ncifcrf.gov/) to explore gene functions and uncover potential therapeutic targets of the active components of SZJN prescription. Analysis of the pathways provided scientific connotation for SZJN prescription.

### Materials and reagents for biological experiments

Differentiated PC12 cells, RPMI-1640 complete culture medium (containing 10% FBS, 100 U/mL penicillin, and 100 U/mL of streptomycin) and RPMI-1640 basic culture medium were purchased from Procell Life Science and Technology Co. Ltd. (Wuhan, China); Caspase-3 primers were synthesized by Sangon Biotechnology Co. Ltd. (Shanghai, China);. L-glutamic acid (glutamate) was purchased from Sigma (USA); SZJN prescription, constituted from Ginseng, Anemarrhenae, and Paeoniae rubra, was acquired from the Chinese Medicine Preparation Department of China-Japan Friendship Hospital; Memantine hydrochloride (MH) was purchased from H. Lundbeck A/S (Denmark). SZJN prescription and glutamate were dissolved in RPMI 1640 complete medium, while MH was dissolved in dimethyl sulfoxide (DMSO) to obtain a final concentration of less than 0.5% for use in the experiments.

### Cell culture and modeling intervention

PC12 cells were cultured in RPMI-1640 complete medium at 95% humidity, 5% carbon dioxide (CO_2_), and a temperature of 37 °C. Cells were treated with or without SZJN prescription (0.05, 0.1 and 0.2 mg/mL), MH (10 μM), and glutamate (22.5 mM) for 24 h. Each experiment was performed in triplicate.

### Cell viability and cell death assays

Real-time examination of cell viability and cell death was achieved using the live-cell Essen Bioscience IncuCyte imaging system, which recorded both phase-contrast and fluorescent images over time. Briefly, cells (30,000 cells/well) were seeded into Corning 96-well tissue culture plates and then placed on the IncuCyte instrument. Results for cell viability and cell death were recorded for over 36 h. Dead cells were identified using fluorescent nuclear dye YOYO-1(Thermo Fisher Scientific, USA), a cell impermeable dye that enters cells via compromised membranes. To calculate the average percentage of cell death, the green fluorescent area (YOYO-1 positive) was divided by the total phase area.

### Cell apoptosis using flow cytometry (FCM)

Apoptotic cells were quantitated using the Annexin V-FITC/propidium iodide (PI) apoptosis assay kit (KeyGEN BioTECH, China). Briefly, PC12 cells were digested with 0.25% trypsin, centrifuged, and resuspended in 200 μL 1 × binding buffer. Then, cells were stained with 5 μL Annexin V-FITC in the dark for 10 min. This was followed by another staining with 5 μL PI for 5 min. Samples were analyzed using a FACScan flow cytometer (BD, USA). At least 10,000 events were recorded. Apoptotic cells were expressed as a percentage of the total number of cells.

### Effect of SZJN prescription on the mRNA expression of Caspase-3 in glutamate-treated PC12 cells

The effect of SZJN prescription on Caspase-3 was established through quantitative real-time polymerase chain reaction (qRT-PCR) analysis. Briefly, total RNA from cells was extracted using the RNA Extraction Kit (KURABO, Japan) according to the manufacturer’s instructions. The concentration and purity of the extracted RNA were determined using a Q5000 nucleic acid protein analyzer (Quawell, USA) . Then a reverse transcription kit (Thermo Fisher Scientific, USA) was used to reverse transcribed the RNA to cDNA. qRT-PCR was performed using the SYBR Green Master Mix on a CFX Connect Real-Time PCR System (Bio-Rad, USA), with Caspase-3: Forward: 5′-GTACAGAGCTGGACTGCGGTATTG-3′; Reverse: 5′-AGTCGGCCTCCACTGGTATCTTC − 3′ and β-actin: Forward: 5′-GCAGTTGGTTGGAGCAA-3′; Reverse: 5′-ATGCCGTGGATACTTGGA-3′ primer pairs. qRT-PCR conditions were as follows: Initial denaturation step at 95 °C for 10 min, followed by 40 cycles of 95 °C for 15 s, 60 °C for 1 min. β-actin served as the internal reference gene. The relative expression levels of target genes were calculated using the 2^−∆∆CT^ method.

### Statistical analysis

All statistical analyses were performed via SPSS version 25.0. Data were presented as means ± standard deviations (SD). Statistical comparisons were achieved using one-way analysis of variance (ANOVA) for multiple groups with variance depicting homogeneous normal distribution data. Least—Significant Difference (LSD) test was applied for mean separations to reveal statistically significant differences, *P* < 0.05.

## Results

### Potential active components of SZJN prescription

Based on the set conditions in the database, 45 potentially active SZJN prescription components were retrieved and screened, including 17, 15, and 13 for Ginseng*,* Anemarrhenae, and Paeoniae rubra, respectively (Table 1).

**Table 1:** Potential active components of Ginseng, Anemarrhenae and Paeoniae rubra

Herb	Mol ID	Molecule Name	MW	OB (%)	DL
Ginseng	MOL005348	Ginsenoside-Rh4_qt	458.8	31.11	0.78
Ginseng	MOL005357	Schisantherin B	514.62	31.99	0.83
Ginseng	MOL005376	Panaxadiol	460.82	33.09	0.79
Ginseng	MOL005344	Ginsenoside Rh2	622.98	36.32	0.56
Ginseng	MOL000358	beta-Sitosterol	414.79	36.91	0.75
Ginseng	MOL005399	alexandrin_qt	414.79	36.91	0.75
Ginseng	MOL004492	Chrysanthemaxanthin	584.96	38.72	0.58
Ginseng	MOL005317	Deoxyharringtonine	515.66	39.27	0.81
Ginseng	MOL005401	Ginsenoside Rg5_qt	442.8	39.56	0.79
Ginseng	MOL002879	Diisooctyl phthalate	390.62	43.59	0.39
Ginseng	MOL000449	Stigmasterol	412.77	43.83	0.76
Ginseng	MOL005384	Suchilactone	368.41	57.52	0.56
Ginseng	MOL005360	Malkangunin	432.56	57.71	0.63
Ginseng	MOL000787	Protopine	353.4	59.26	0.83
Ginseng	MOL003648	(+)-Maackiain	284.28	65.83	0.54
Ginseng	MOL005321	Frutinone A	264.24	65.9	0.34
Ginseng	MOL005314	Celabenzine	379.55	101.88	0.49
Anemarrhenae	MOL001677	Aurantiamide acetate	444.57	58.02	0.52
Anemarrhenae	MOL003773	Mangiferolic acid	442.75	36.16	0.84
Anemarrhenae	MOL000422	Kaempferol	286.25	41.88	0.24
Anemarrhenae	MOL004373	Icaritin	368.41	45.41	0.44
Anemarrhenae	MOL004489	Anemarsaponin F_qt	432.71	60.06	0.79
Anemarrhenae	MOL004492	Chrysanthemaxanthin	584.96	38.72	0.58
Anemarrhenae	MOL004497	Hippeastrine	315.35	51.65	0.62
Anemarrhenae	MOL004514	Timosaponin B III_qt	416.71	35.26	0.87
Anemarrhenae	MOL000449	Stigmasterol	412.77	43.83	0.76
Anemarrhenae	MOL004528	Icariin	676.73	41.58	0.75
Anemarrhenae	MOL004540	Anemarsaponin C_qt	416.71	35.5	0.87
Anemarrhenae	MOL004542	Anemarsaponin E_qt	448.76	30.67	0.86
Anemarrhenae	MOL000483	N-cis-FeruloyltyraMine	313.38	118.35	0.26
Anemarrhenae	MOL000546	Diosgenin	414.69	80.88	0.81
Anemarrhenae	MOL000631	cis-N-p-Coumaroyltyramine	283.35	112.9	0.2
Paeoniae rubra	MOL001910	11alpha,12alpha-Epoxy-3beta,23-dihydroxy-30-nor-olean-20(29)-en-28,13beta-olide	470.71	64.77	0.38
Paeoniae rubra	MOL001918	Paeoniflorgenone	318.35	87.59	0.37
Paeoniae rubra	MOL001919	Palbinone	358.52	43.56	0.53
Paeoniae rubra	MOL001921	Lactiflorin	462.49	49.12	0.8
Paeoniae rubra	MOL001924	Paeoniflorin	480.51	53.87	0.79
Paeoniae rubra	MOL001925	Paeoniflorin_qt	318.35	68.18	0.4
Paeoniae rubra	MOL001928	Albiflorin_qt	318.35	66.64	0.33
Paeoniae rubra	MOL001930	Benzoylpaeoniflorin	584.62	31.27	0.75
Paeoniae rubra	MOL000211	Betulinic acid	456.78	55.38	0.78
Paeoniae rubra	MOL000358	beta- Sitosterol	414.79	36.91	0.75
Paeoniae rubra	MOL000359	Sitosterol	414.79	36.91	0.75
Paeoniae rubra	MOL000422	Kaempferol	286.25	41.88	0.24
Paeoniae rubra	MOL000492	(+)-Catechin	290.29	54.83	0.24

### Predicted targets for SZJN prescription chemical components, VD significant and therapeutic targets

**Fig. 2:**
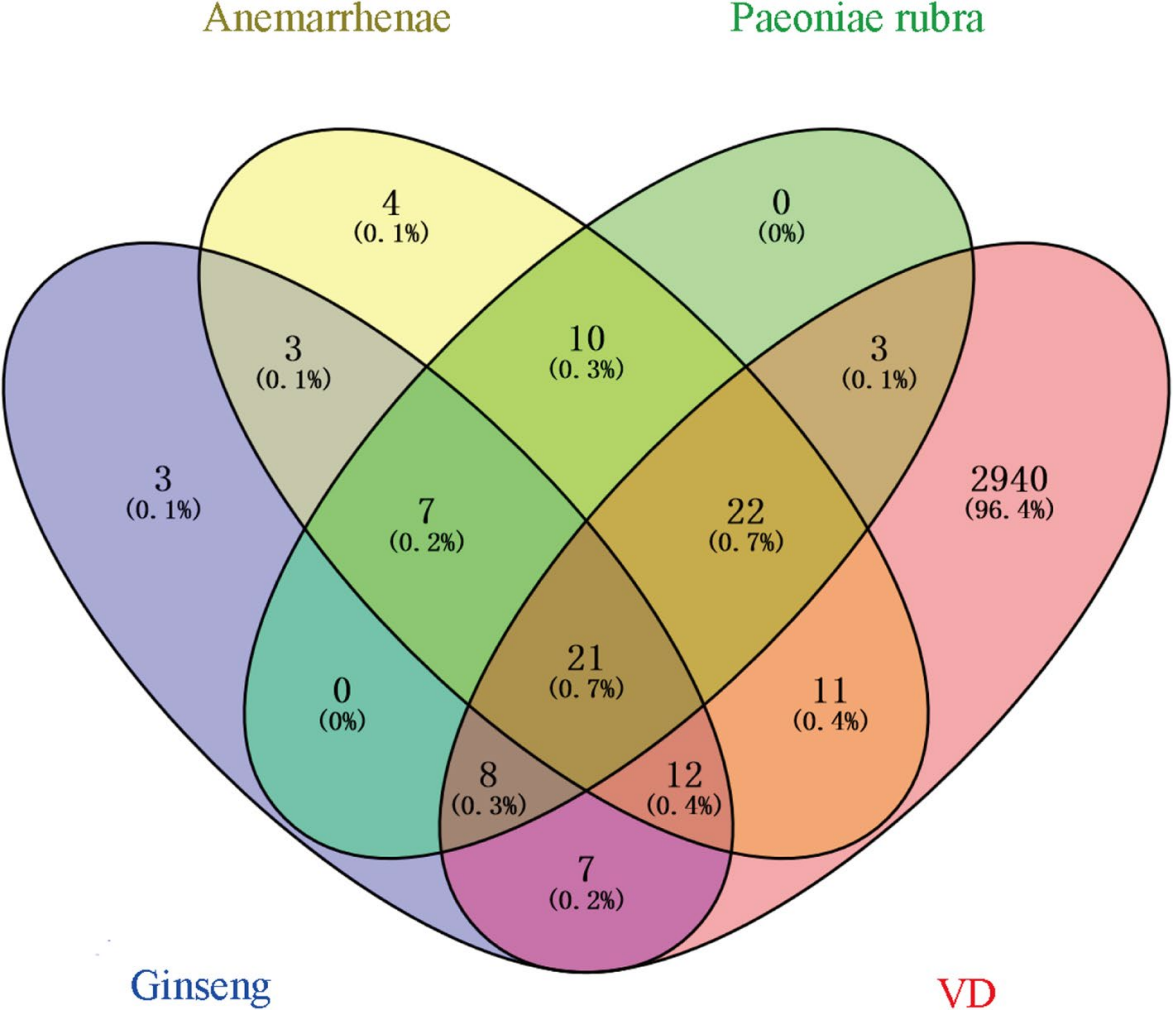
The overlapped targets between SZJN prescription potential targets and VD targets. Venn diagram demonstrates the number of shared and unique targets by SZJN prescription and VD

Names of component targets in SZJN prescription were corrected using the UniProt database, after which repeated targets were eliminated. Eventually, 61, 90, and 71 potential targets for Ginseng, Anemarrhenae, and Paeoniae rubra respectively, were obtained, respectively. From the GeneCards and DisGeNET databases successfully revealed all genes related to VD were revealed; 3077 target genes were retrieved. Integrating potential targets of chemically active components in SZJN prescription with those of VD revealed exactly 21 therapeutic targets (Fig. 2).

### Network construction and analysis

**Fig. 3:**
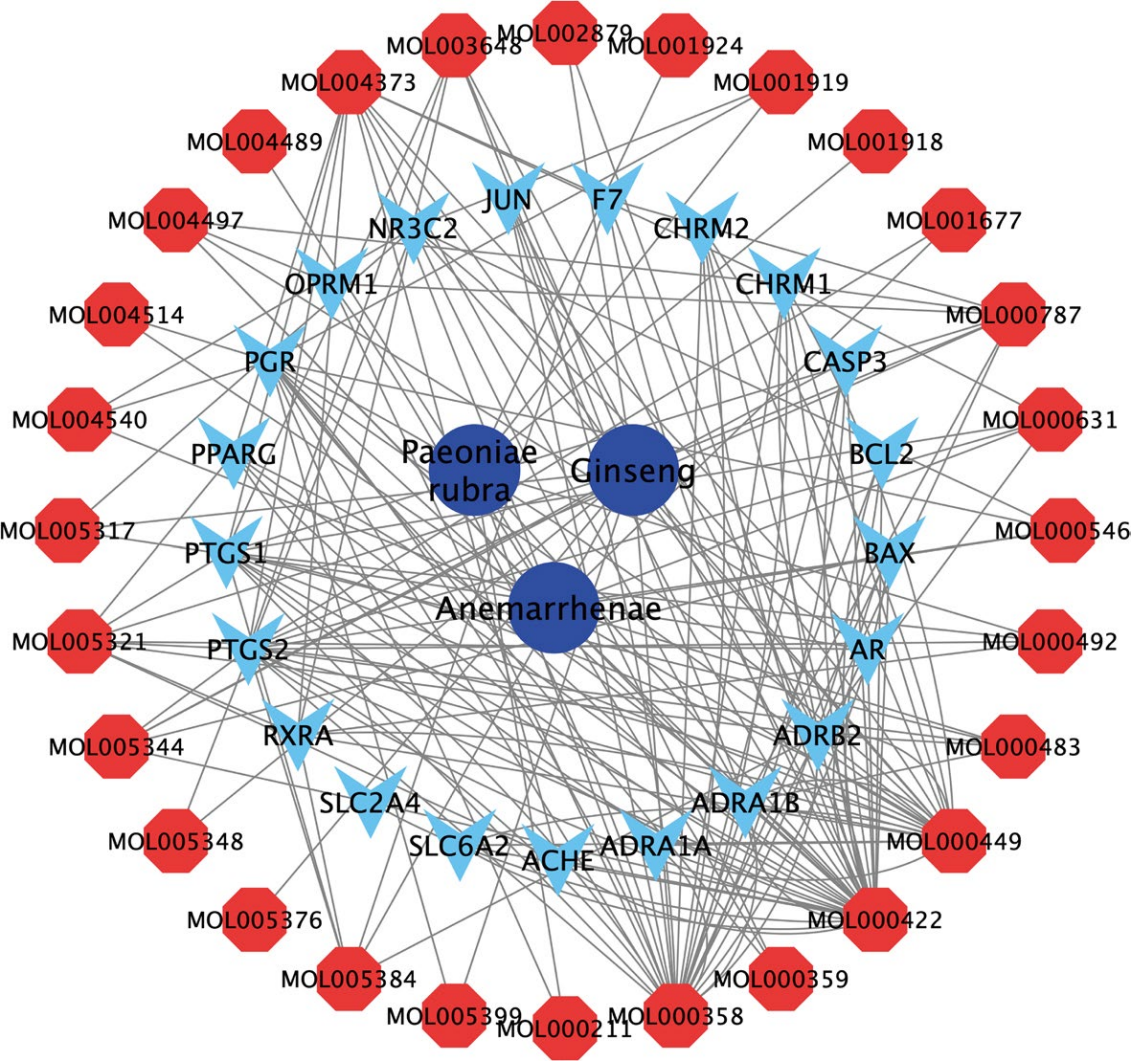
A network of SZJN prescription-active compounds-VD targets. Dark blue nodes stand for herbs of SZJN prescription, red nodes stand for active compounds, light blue nodes represent the shared targets between SZJN prescription potential targets and VD targets

A network of SZJN prescription-active compounds-VD targets was successfully constructed via Cytoscape 3.7.1 for topological analysis (Fig. 3). It comprised 52 nodes and 183 edges; a dark blue node represented single herb (3), a red node depicted active compounds (28), and a light blue node denoted therapeutic targets (21).

**Fig. 4:**
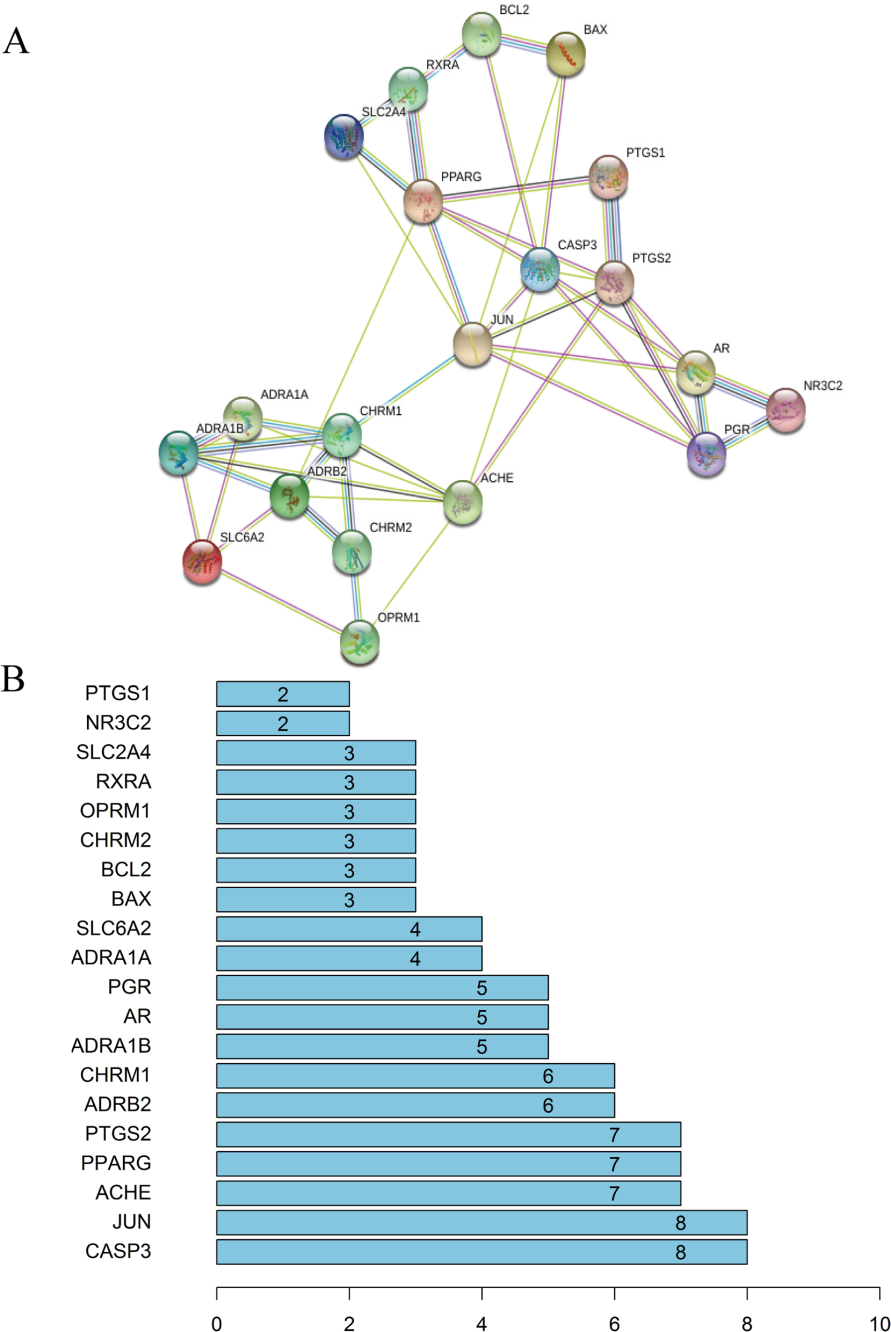
Topological analysis of the target proteins of VD and SZJN prescription. **a** PPI network diagram of the common targets of VD and SZJN prescription. **b** Bar graph of all protein nodes degree related to the targets

Twenty-one therapeutic SZJN prescription targets were input into the STRING software and identified 20 target proteins under the set conditions. These results were saved as a TSV file. Network topology analysis and calculations were performed via Cytoscape 3.7.1, for the construction of a PPI network (Fig. 4a). Based on the degree of the nodes, a bar graph depicting the relationship between all protein nodes and the targets was generated (Fig. 4b).

### GO and KEGG pathway enrichment analyses

**Fig. 5:**
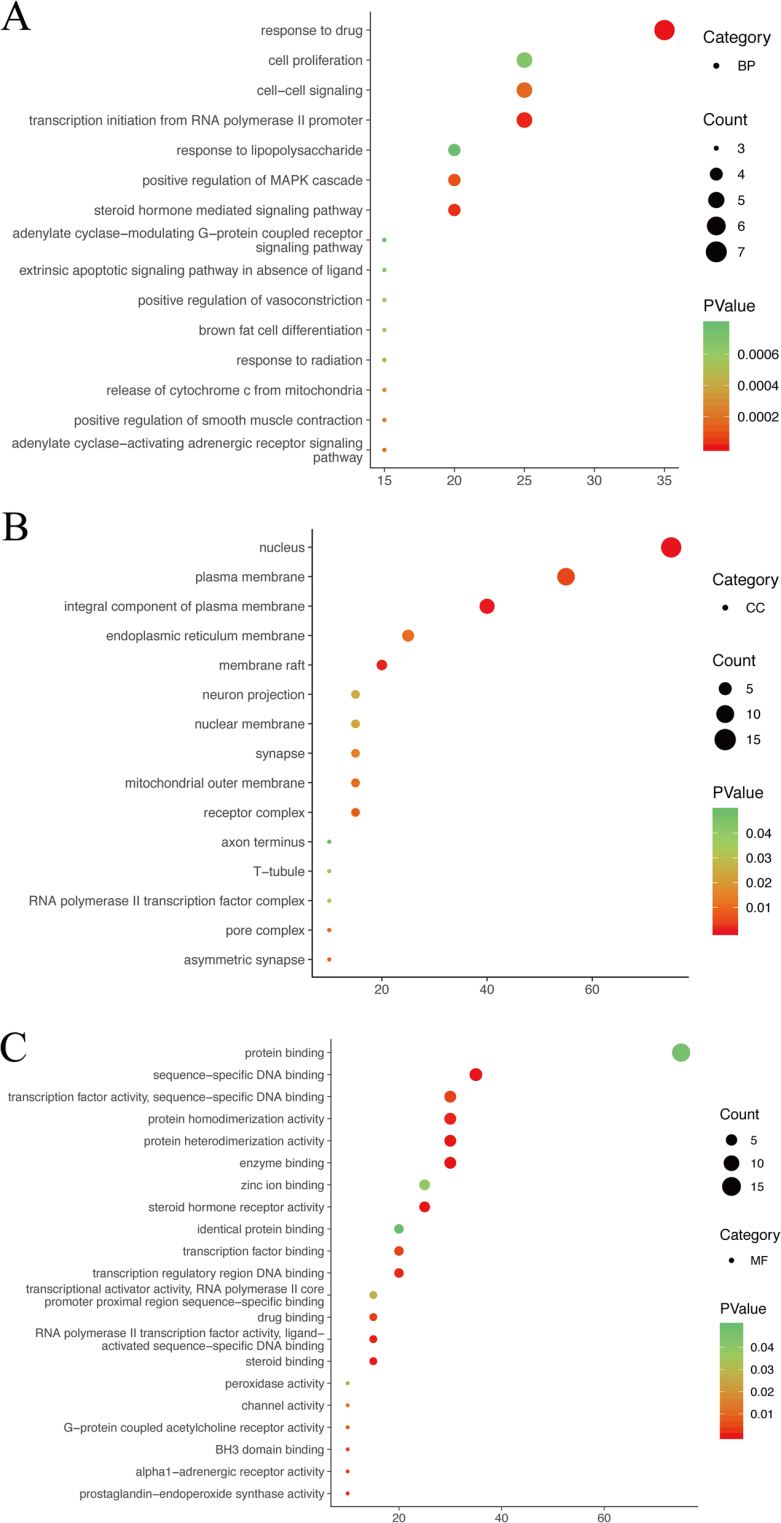
Bubble chart showing GO enrichment analyses. **a** GO-BP enrichment analysis. **b** GO-CC enrichment analysis. **c** GO-MF enrichment analysis

Through GO enrichment analysis of the 20 target proteins in the PPI network, 106 GO items were revealed (*P* < 0.05), including 70 items related to biological processes, such as drug reaction, transcription initiation of RNA polymerase II promoter, and steroid mediated signaling pathway; 15 items related to cell composition, such as nuclear and plasma membrane components, and membrane raft; 21 items related to molecular function, (steroid receptor, sequence-specific DNA binding, and enzyme binding) were identified (Fig. 5a-c).

**Fig. 6:**
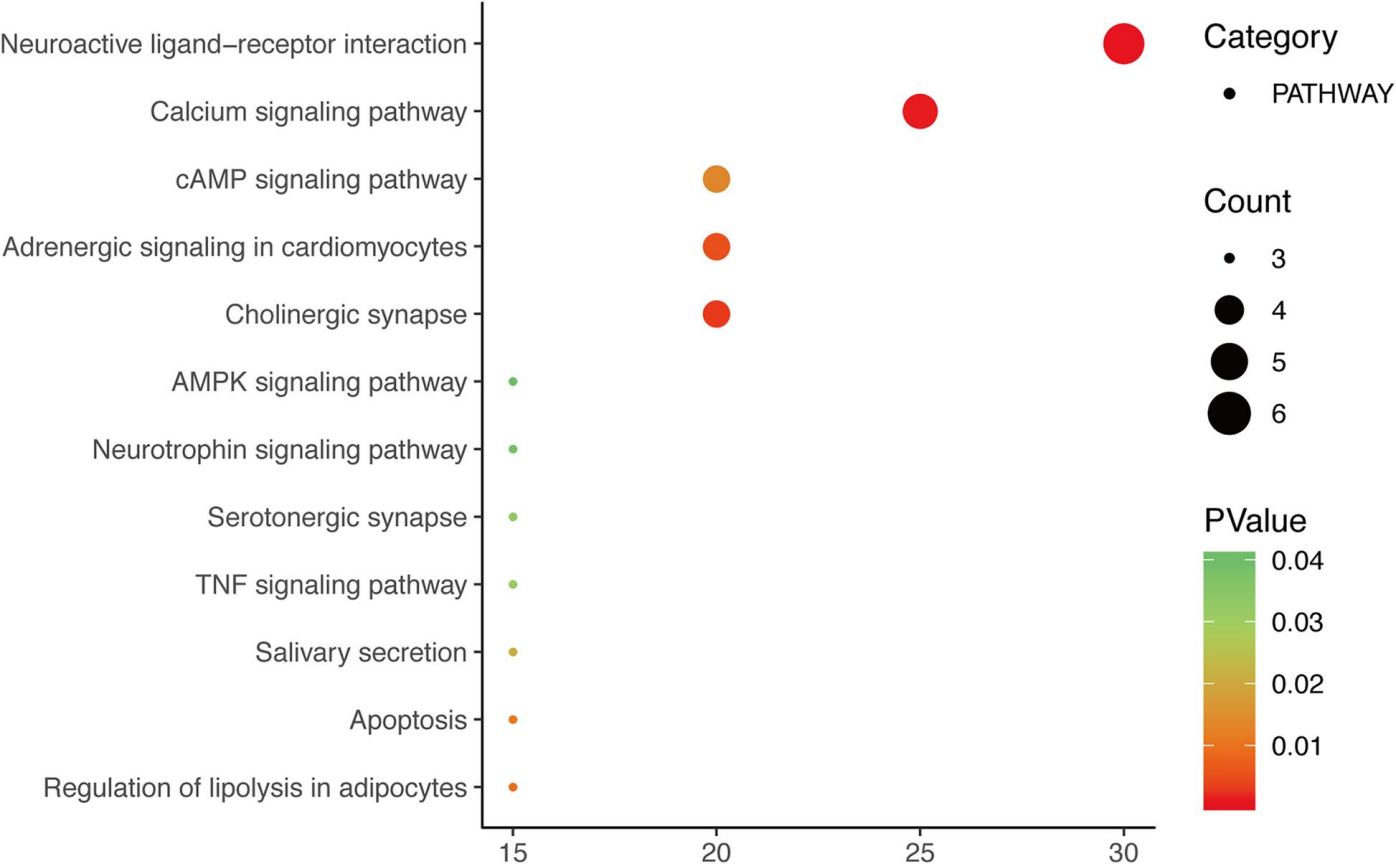
Bubble chart of KEGG pathway analysis

**Fig. 7:**
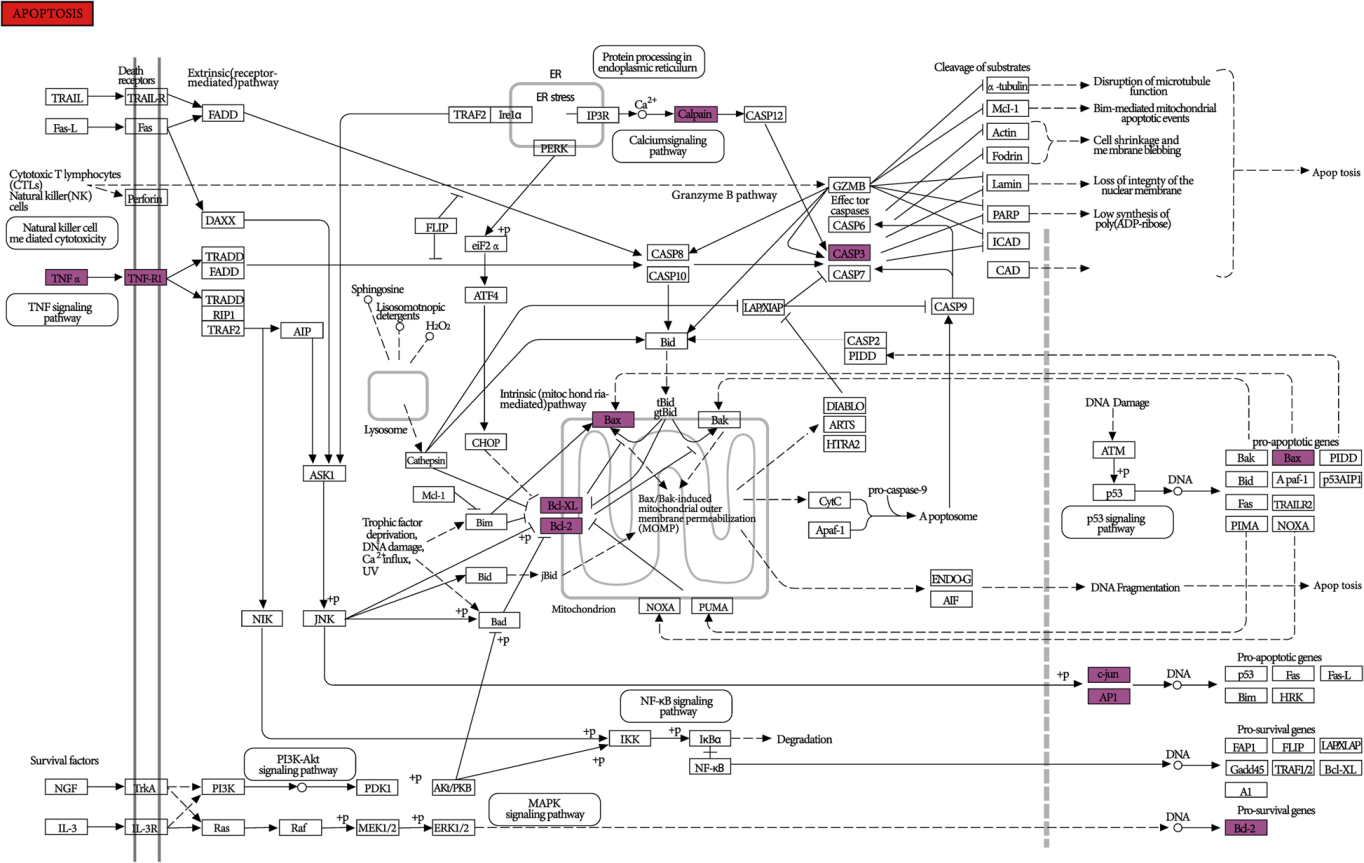
Pathway map of SZJN prescription against VD. The key targets of SZJN prescription in the treatment of VD are shown purple in the apoptosis pathway. Arrows represent the activation effect, T-arrows represent the inhibition effect, and segments show the activation effect or inhibition effect

Also, KEGG pathway analysis of the 20 target proteins in the PPI network was performed (at *P* < 0.05), and the unrelated pathways including those for cancer and hepatitis B were eliminated. Results demonstrated that the targets of SZJN prescription for treating VD treatment were mainly associated with neuroactive ligand-receptor interaction, calcium, cholinergic synapse, cardiac myocyte adrenergic signal transduction, adipocyte lipolysis regulatory, apoptosis, cAMP, salivary secretion, tumor necrosis factor, 5-hydroxy color Aminergic synaptic, neurotrophin and AMPK signaling pathways (Fig. 6). The SZJN prescription-related targets and pathways associated with apoptosis-mediated VD progression have been presented in Fig. 7; the pathway targets are marked in white, whereas the targets of SZJN prescription for VD treatment are marked in purple. It was suggested that SZJN prescription potentially regulates apoptosis, therefore, is crucial in VD treatment.

### Validation of the neuroprotective effects of SZJN prescription against glutamate-induced PC12 cell death

**Fig. 8:**
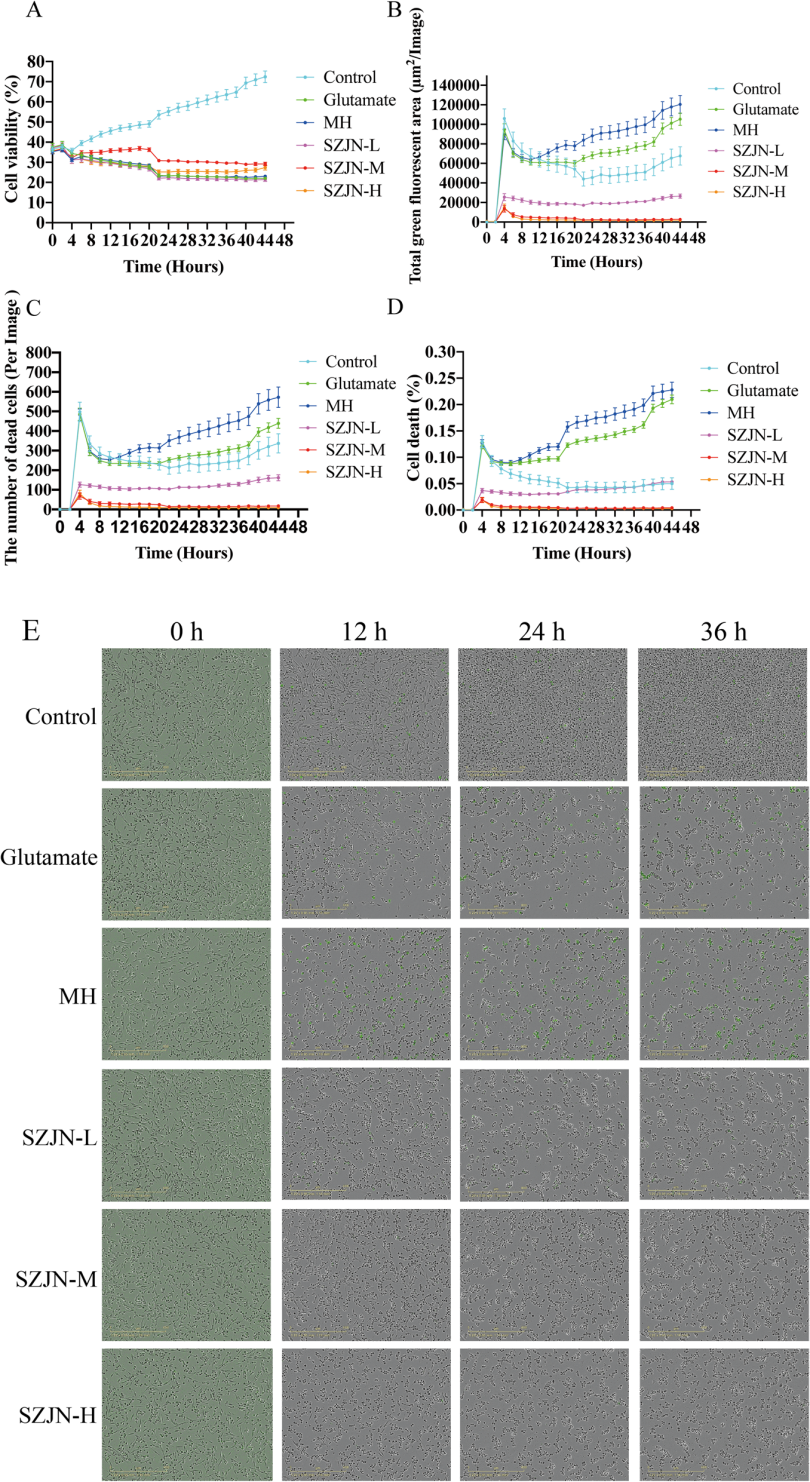
Neuroprotective effects of SZJN prescription against glutamate-induced PC12 cell death. Cells were induced by glutamate solution (22.5 mM) and treated with SZJN prescription (0.05, 0.1 and 0.2 mg/mL) and MH (10 μM) containing the fluorescent dye YOYO-1 to identify dead cells for over 24 h using IncuCyte instrument (*n* = 3). Cells were imaged every 2 h for 24 h on the Essen Bio Science IncuCyte. **a** Time course cell viability analysis of PC12 cells using IncuCyte instrument. **b-d** Time course evaluation of total green fluorescent area, the number of dead cells and percent cell death over 24 h by YOYO-1 uptake on an IncuCyte instrument. **e** Representative images of YOYO-1 positive cells at 0, 12, 24, 36 hpt

The results from the network pharmacological analysis demonstrated that cell proliferation, gene transcription, and cell-cell signaling, and apoptosis were associated with multiple synergies of SZJN prescription on VD treatment. The cytotoxicity of glutamate in PC12 cells was evaluated using YOYO-1 and then the neuroprotective effects of SZJN prescription against glutamate-induced cell death were validated. Cells treated with 22.5 mM glutamate exhibited low viability (Fig. 8a) and showed a large total green fluorescence area within 4 h post-treatment (hpt). The fluorescence intensity increased almost 10 times by 36 hpt (Fig. 8b). Similarly, the number of dead cells cultured with glutamate increased by 4 hpt. Death cell counts increased to over 400/image by 24 hpt (Fig. 8c). The average percent cell death recorded every 2 h over 36 h of monitoring for PC12 cells injury is presented in Fig. 8d. Glutamate-induced cells exhibited a significantly high death rate over the time course, approximately 20% after 36 h of treatment. For injured cells, with complete media, less than 5% cell death was reported over the monitoring time. Exposure to glutamate (22.5 mM) triggered cell death, however, this effect was reversed by SZJN prescription (0.05, 0.1, and 0.2 mg/mL). Moreover, SZJN prescription reduced the total green fluorescence, lowered the number of dead cells and the average percentage of cell death, and recovered cell viability (Fig. 8a-d). Microscopic images were analyzed for positive cell nuclei based on the size and fluorescence intensity of YOYO-1. Representative images of YOYO-1 positive cells at 0, 12, 24, 36 hpt are presented in Fig. 8e.

### SZJN prescription prevented glutamate-induced PC12 cell death through apoptosis

**Fig. 9:**
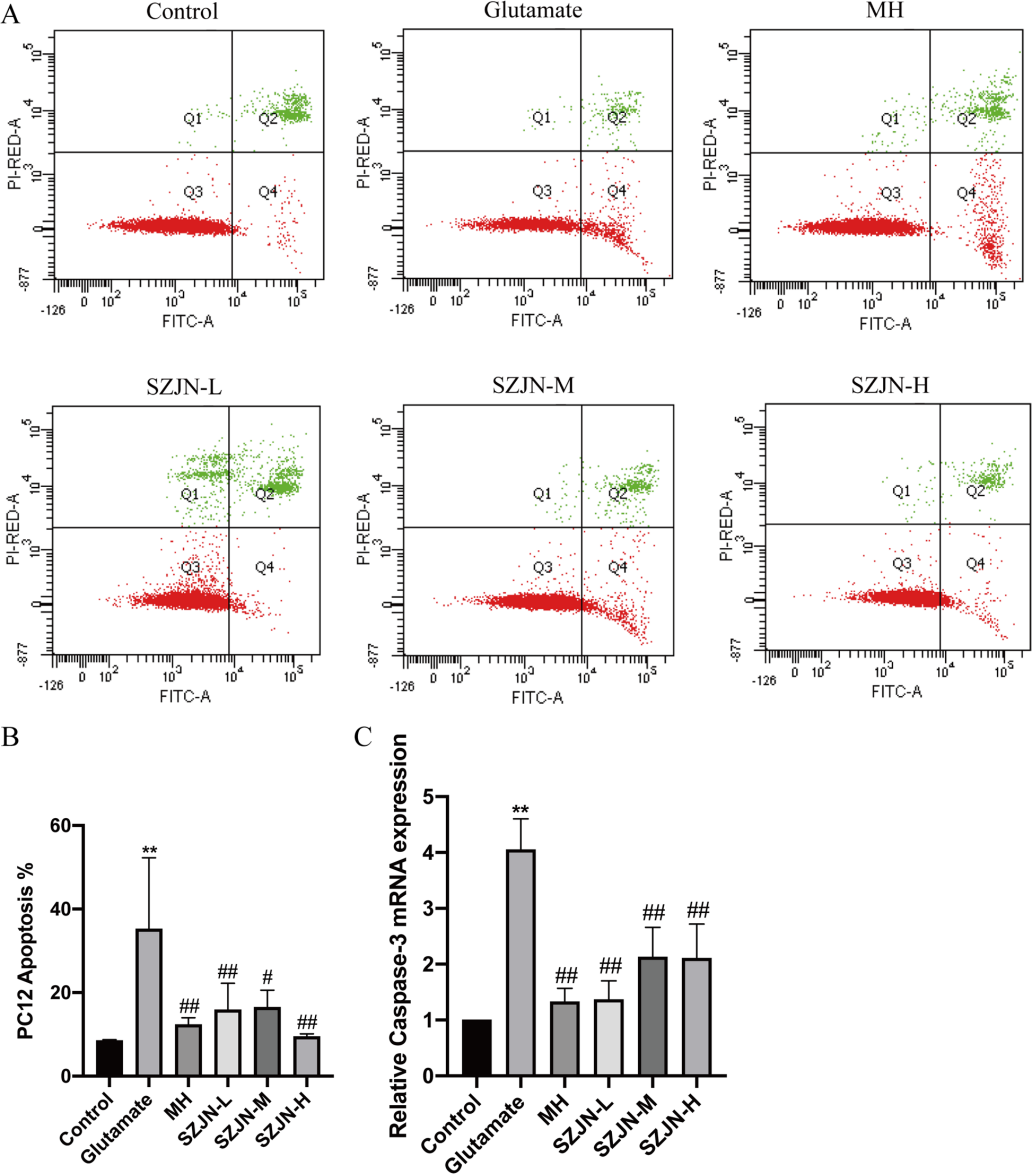
SZJN prescription inhibited cell death through Apoptosis. **a** Cell apoptosis measured by Annexin V-FITC/PI staining. **b** Statistical columns of apoptotic cells. **c** Relative expression of caspase-3 mRNA in each group. The data were expressed as means ± SD (*n* = 3). ^*^P<0.05, ^**^P<0.01 showed significant difference compared with the control group (Control). ^#^*P*<0.05, ^##^*P*<0.01 showed significant difference vs the glutamate-induced group (Glutamate)

Annexin V-FITC/PI double staining was employed to examine apoptotic cells and verify the preventive effects of SZJN prescription on glutamate-induced neurotoxicity through inhibition of cell apoptosis (Fig. 9a). In Fig. 9b, 35.23% ± 17.03% of cells treated with glutamate for 24 h were detected in the apoptotic stage and were significantly higher than the control group (8.53% ± 0.21%, *P* < 0.01). However, SZJN prescription treatment (0.05, 0.1, and 0.2 mg/mL) and MH significantly decreased the number of apoptotic cells to 15.87% ± 6.37%, 16.47% ± 4.13%, 9.47% ± 0.67% and 12.30% ± 1.67%, respectively (*P* < 0.05). The results demonstrated that SZJN prescription inhibited glutamate-induced apoptosis of PC12 cells.

Expression profiles of Caspase-3 mRNA were detected using qRT-PCR. Notably, the relative levels of Caspase-3 mRNA were significantly higher after treatment with glutamate (4.05 ± 0.55) compared to the control group (1.00 ± 0.00, *P* < 0.01). Caspase-3 expression in the SZJN prescription group showed significantly varying profiles as follows: low (SZJN-L, 1.37 ± 0.33, *P* < 0.01), middle (SZJN-M, 2.13 ± 0.53, *P* < 0.01) and high (SZJN-H, 2.11 ± 0.61, *P* < 0.01) relative to the glutamate group. The expression levels in the SZJN-L group exhibited the highest decreasing trend, whereas the expression of Caspase-3 mRNA in the MH group decreased significantly (1.33 ± 0.24, *P* < 0.01) (Fig. 9c).

## Discussion

According to the theory of traditional Chinese medicine (TCM), VD is described as “dementia” or “stroke combined with dementia”. The pathogenesis of VD in TCM is related to “a deficiency of essential Qi with toxin damaging brain collaterals”. Since VD lasts longer, blockage of the Qi and blood may induce cognitive impairment because brain neurons get limited nutrients [17]. Therefore, approaches that improve the essential energy are vital in the treatment of VD. They can also, detoxify and unblock the collaterals. Consequently, the Qi and blood eliminate the accumulated toxigenic products thereby, establishing SZJN prescription.

Previous studies have revealed that several TCM components exert their therapeutic effects by acting on multiple targets [18–21]. For instance, Ginseng was found to improve intelligence and relaxation of the nerves, while ginsenosides have the potential to prevent cerebral ischemia and repair neuron damage, which consequently improves vascular cognitive impairment [22, 23]. Similarly, studies have reported that Anemarrhenae-derived saponins improve cerebral ischemia and hypoxia; this subsequently enhances neurotrophic cognitive ability [24, 25]. Additional reports have demonstrated that Paeoniae rubra potentially protects neurons from hypoxia- or neurotoxicity-derived injury, and reduces inflammatory response [26, 27]. A combination of various herbs in SZJN prescription restore blood collaterals by eliminating phlegm, blood stasis, and collateral toxins. Herbal combinations can strengthen the vital energy and tonify the deficiency, detoxify and unblock collaterals, reduce turbidity and benefit wisdom. In the present study, 28 active compounds in SZJN prescription, 222 compound-related targets, and 3077 VD-related targets were revealed from the public databases. In details, the degree of these three herbs was as follows: Ginseng (12), Anemarrhenae (11), and Paeoniae rubra (8), consistent with their compatibility with SZJN prescription. Ginseng is regarded as the sovereign medicine, whereas Anemarrhenae and Paeoniae rubra are considered minister and assistant medicines, respectively [28]. Thus, it may be speculated that Ginseng had a promising potential in the treatment of VD.

A total of 28 active compounds after eliminating the duplicates were identified, particularly, stigmasterol, beta-sitosterol (β-sitosterol), and kaempferol exhibited obvious bioactivities. Pharmacological studies have confirmed that stigmasterol, β-sitosterol and kaempferol are active components of Ginseng [29, 30]. Moreover, these compounds have been revealed as chemical components of Anemarrhenae and Paeoniae rubra [31, 32]. Stigmasterol can significantly relieve the vascular inflammation and nerve damage through reducing NO levels, COX-2 expression and apoptotic responses [33, 34]. In particular, stigmasterol and β-sitosterol were reported to significantly improve vanadium-induced cognitive ability in mice and were associated with improved motor coordination potential and a reduction in anti-oxygen free radicals and oxidative stress [35]. It has been found that β-sitosterol can lower the cholesterol levels, enhance the production of plasminogen activators, and prevent endothelial dysfunction [36]. β-sitosterol plays a vital role in the direct endothelial protective and antiatherogenic effects against oxidized low-density lipoprotein (ox-LDL) [37]. Besides, β-sitosterol was found to potentially reduce neuron demyelination [38]. Kaempferol has been reported to alleviate ox-LDL-induced apoptosis of human endothelial cells and potentiate angiogenic functions in cultured endothelial cells [39, 40]. Moreover, kaempferol was revealed to improve the damage of striatal neurons and increase levels of tyrosine hydroxylase (TH) and postsynaptic density protein 95 (PSD95) in mice striata. This mechanism aided in anti-neuroinflammation and maintenance of the integrity of the blood-brain barrier (BBB) [41]. These findings demonstrate that the aforementioned compounds are associated with several important activities for VD treatment. Thus, they provide a basis for in-depth research on their active ingredients in SZJN prescription.

With regards to target points, PTGS2, PTGS1, PGR, ADRB2, ADRA1B, and CHRM1 recorded degrees≥10. However, PTGS2 had a maximum degree of 17, an implication that it is a potential SZJN prescription target vital in the management of VD. PTGS2, also known as cyclooxygenase-2 (COX-2), is a subtype of prostaglandin G/H synthetase (PGHS), and a vital enzyme in fatty acid metabolism [42]. PTGS2 overexpression results in endothelial apoptosis, thus mediating vascular endothelial injury [43]. Downregulation of PTGS2 expression could inhibit apoptosis and promote proliferation, migration and angiogenesis of endothelial progenitor cells (EPCs) [44]. Similarly, PTGS1 (COX-1), another subtype of prostaglandin G/H synthetase, has been described as an important potential target for VD treatment. This enzyme protects gastrointestinal mucosa, promotes platelet aggregation, and induces peripheral vascular resistance through regulation of prostacyclin synthesis, closely associated with thrombosis [45–47]. PTGS1 has clear vascular protective roles associated with the anti-thrombotic potential, vasoconstriction and the development of atherosclerosis and vascular inflammation [48]. On the other hand, the steroid hormone PGR, potentially reduces oxidative stress and neuroinflammation, regulates mitochondrial function, and promotes myelination by triggering several signaling pathways, such as the MAPK and NF-κ B signaling pathways. Therefore, it exerts a therapeutic effect on nervous system injury and neurodegenerative diseases [49]. ADRB2, a transmembrane β-adrenergic receptor, interacts with adrenaline (a hormone and neurotransmitter) to mediate muscle and nerve function through downstream interaction with L-type calcium channels [50]. Moreover, it has been demonstrated that activation of ADRB2 significantly increased EPCs bioactivities including cell proliferation, migration, and tube formation abilities [51]. However, downregulation of ADRB2 could induce the dysfunction of EPCs. There are reports on the critical role of CHRM1 in memory impairment, and a decrease in its density is associated with learning and memory disorder, and cognition dysfunction [52]. Researchers speculate that SZJN prescription may target the aforementioned proteins by regulating their expression levels to promote VD treatment. However, information on this relationship remains scarce and warrants further exploration.

The topology of the PPI network demonstrated that CASP3, JUN, ACHE, PPARG, PTGS2, ADRB2, CHRM1, ADRA1B, AR, PGR (degree≥4.7) are potentially important targets for SZJN prescription in VD treatment. This is based on its multi-target, multi-path, and multi-system characteristics. However, all the targets in the network, except for PTGS2, were consistent with the PPI network. This controversy in results may be attributed to the scarcity of information regarding whether the herbs were processed or decocted, as well as the specificity of target action. It was found that CASP3 recorded the largest degree (8), therefore it acted as the main target for SZJN prescription. Caspase-3, a member of the cysteine protease family, is a downstream protein in the apoptosis pathway and an apoptotic enzyme that functions through the death receptor and mitochondrial pathways [53]. The apoptosis process may be attributed to the delayed neuronal degeneration of the central nervous system following injury to the distal axon [54]. Previous studies found a significant increase in Caspase-3 expression in the hippocampus of VD rats [55–58], which demonstrated that Caspase-3-mediated apoptosis may be an important molecular mechanism of VD [59, 60]. Herein, the treatment with SZJN prescription showed protective activity against glutamate-induced PC12 cell apoptosis. Similar reports have previously been published, whereby ginsenosides in SZJN prescription protected neural cells against glutamate-mediated oxidative stress and neuronal cell death [61, 62]. Moreover, a significant increase in the relative expression of Caspase-3 mRNA in PC12 cells was found after glutamate injury, consistent with reports from previous studies [63, 64]. This expression was, however, down-regulated with low, middle and high doses of SZJN prescription. Besides, MH significantly reduced the levels of Caspase-3 expression; this consequently inhibited cell apoptosis and protected the nerve cells. With these findings, SZJN prescription potentially lowers Caspase-3 expression and the apoptosis of neurons as it targets the Caspase-3. This is considered to be a vital step in the treatment of VD. Other targets, include JUN, a transcription factor for AP-1 that is closely linked to neuronal death caused by a β amyloid protein [65], this factor potentially activates the downstream apoptotic pathway.

In the present study, following GO functional enrichment analyses, it was revealed that target genes were associated with a drug reaction, transcription initiation of RNA polymerase II promoter, cell signal transduction, and cell proliferation, among others. These observations demonstrated that SZJN prescription may promote cell proliferation and reduce cell apoptosis. Results from related experiments indicate that SZJN prescription mediates the repair of damaged nerve cells via different mechanisms [66, 67].

Moreover, the KEGG metabolic pathway analysis revealed that the targets were associated with neuroactive ligand-receptor interaction, calcium, and cholinergic synapse signaling pathways. Of note, the neuroactive ligand-receptor interaction signaling pathway incorporates all receptors and ligands related to intracellular and extracellular signaling pathways in the plasma membrane. These include adrenergic, muscarinic acetylcholine receptor (mAChR), and dopamine receptors, among others [68]. Studies have attributed the abnormal expression of various receptor genes in the signaling pathway of receptor interaction in neuroactive ligands to a decline in learning and memory ability, and cognitive dysfunction [69–71]. Many subtypes of the adrenergic receptors, such as ADRB2, ADRA1A, ADRA1B, have been described, whereby a combination of these receptors and norepinephrine were found to regulate cell energy metabolism, glutamate transport, and neuroinflammatory response. These reports suggest that the adrenergic receptors represent a vital pharmacological target for the recovery and enhancement of cognitive function [72]. More studies have shown that muscarinic acetylcholine receptor (mAChR), an important neurodegenerative disease drug target, is linked to neuronal excitability, synaptic plasticity, acetylcholine release, and cognitive function, and possesses anti-apoptosis characteristics [73]. In this study, ADRB2, ADRA1A, ADRA1B, and CHRM1 were considered as SZJN prescription targets, and may exert a pharmacological effect in the management of VD through the interaction between ligands and receptors.

Due to the close relationship exists between the Ca^2+^ signaling pathway and signal transmission of neuron synapses, it was found that an open voltage-gated Ca^2+^ channel could activate NMDA receptors and drive the release of neurotransmitters. Also, an imbalance in the Ca^2+^ signaling pathway potentially activated apoptosis signaling molecules, such as Caspase-3 and Bax. This process inhibits the expression of Bcl-2 and consequently activates the apoptosis pathway [74]. Another report revealed significantly high calcium concentration in the primary hippocampal neurons following ischemia-reperfusion injury. This led to calcium overload, and subsequently activation of the apoptosis signaling pathway, which eventually induced neuron apoptosis [75]. In the present study, Caspase-3, Bax, and Bcl-2 were targets of SZJN prescription, therefore, had an association with the calcium signaling pathway. SZJN prescription exerted potential regulatory effects on the 3 targets via the calcium signaling pathway; this could be vital in the pharmacological treatment of VD.

Furthermore, the central cholinergic system coordinates synaptic plasticity, learning, and memory ability. Acetylcholine (ACh) is a neurotransmitter of the cholinergic pathway, and functions as a nicotinic (N) or muscarinic cholinergic receptor (CHRM1–5). Notably, excess hydrolysis of ACh by AChE has been implicated in the loss of cholinergic neurons, prolonged synaptic signal transmission disorder, and cognitive dysfunction [76]. Inhibition of AChE and activation of the cholinergic system have been reported to alleviate the central inflammatory response and repair the synaptic function [77]. This study reported AChE, CHRM1, and CHRM2 as targets for SZJN prescription, as they exerted potential effects via the cholinergic system. Therefore, it was hypothesized that SZJN prescription may regulate AChE, CHRM1, and CHRM2 via the cholinergic signaling pathway, which is critical in the pharmacological treatment of VD. Several other genes and metabolic pathways, as revealed by GO functional enrichment and KEGG metabolic pathways, may also be targets for active components of SZJN prescription. An in-depth analysis of these factors is needed to elucidate their potential molecular mechanisms in SZJN prescription.

However, there are still some limitations for this research. For example, the network pharmacology research relies more on various existing databases, but the screening criteria of compounds in databases may not be accurate enough. Moreover, the screened active ingredients might be inconsistent with the ingredients actually absorbed in blood of the patients with VD. As the accuracy and reliability of data has a great impact on the prediction results, it is necessary to further verify the reliability and quality of the predicted results, optimize the screening criteria of TCM databases, and improve the network pharmacology research. Therefore, further experimental and clinical verification of the efficacy of SZJN prescription on cerebrovascular microenvironment and neuroprotection is demanded to validate theoretical predictions.

## Conclusions

In this study, a systematic analysis for the characteristic of “multicomponents, multitargets, and multichannels” of SZJN prescription in treating VD was successfully performed through the method of network pharmacology and in vitro experiments. The experimental results were in good accordance with the results predicted from the network analysis of the SZJN prescription, especially the anti-apoptosis properties of the prescription. In the future, more experimental evidence should be provided for the neuroprotective and cerebral vascular protective effects of SZJN prescription against VD according to the results of network pharmacology research.

## Data Availability

The data that support the findings of this study are available from the corresponding author upon reasonable request.

## Abbreviations

AChE: Acetylcholinesterase
AMPK: Adenosine 5′-monophosphate (AMP)-activated protein kinase
ADRA1A: Adrenergic receptor α 1A
ADRA1B: Adrenergic receptor α 1B
ADRB2: Adrenoceptor Beta 2
CASP3: Caspase-3
CHRM1: Muscarinic cholinergic receptor M1
CHRM2: Muscarinic cholinergic receptor M2
cAMP: Cyclic adenosine monophosphate
GO: Gene Ontology
JUN: Transcription factor AP-1
KEGG: Kyoto Enrichment of Genes and Genomes
NMDAR1: N-methyl-d-aspartic acid receptor 1
PPI network: Protein-Protein Interaction network
PTGS2: Prostaglandin-endoperoxide synthase 2
PTGS1: Prostaglandin-endoperoxide synthase 2
PGR: Progesterone receptor
PPARG: Peroxisome proliferator-activated receptor-gamma
qRT-PCR: Quantitative real-time polymerase chain reaction
SZJN prescription: Shenzhi Jiannao prescription
TCMSP: Traditional Chinese Medicine Systems Pharmacology
VD: Vascular dementia
